# Evaluation of a SUMO E2 Conjugating Enzyme Involved in Resistance to *Clavibacter michiganensis* Subsp. *michiganensis* in *Solanum peruvianum*, Through a Tomato Mottle Virus VIGS Assay

**DOI:** 10.3389/fpls.2015.01019

**Published:** 2015-12-17

**Authors:** Mayra J. Esparza-Araiza, Bernardo Bañuelos-Hernández, Gerardo R. Argüello-Astorga, José P. Lara-Ávila, Paul H. Goodwin, María I. Isordia-Jasso, Rosalba Castillo-Collazo, Alejandra Rougon-Cardoso, Ángel G. Alpuche-Solís

**Affiliations:** ^1^División Biología Molecular, Instituto Potosino de Investigación Científica y Tecnológica A.C.San Luis Potosí, México; ^2^Facultad de Agronomía y Veterinaria, Universidad Autónoma de San LuisSan Luis Potosí, México; ^3^School of Environmental Sciences, University of GuelphGuelph, ON, Canada; ^4^Laboratory of Agrogenomic Sciences, Universidad Nacional Autónoma de México, ENES-LeónLeón, México

**Keywords:** SUMO-conjugating enzyme, virus-induced gene-silencing, Tomato Mottle Virus, bacterial canker, *Clavibacter michiganensis* subsp. *michiganensis*

## Abstract

*Clavibacter michiganensis* subsp. *michiganensis* (*Cmm*) causes bacterial wilt and canker of tomato. Currently, no *Solanum lycopersicum* resistant varieties are commercially available, but some degree of *Cmm* resistance has been identified in *Solanum peruvianum*. Previous research showed up-regulation of a SUMO E2 conjugating enzyme (*SCE*I) transcript in *S. peruvianum* compared to *S. lycopersicum* following infection with *Cmm*. In order to test the role of *SCE*I in resistance to *Cmm*, a fragment of *SCE*I from *S. peruvianum* was cloned into a novel virus-induced gene-silencing (VIGS) vector based on the geminivirus, Tomato Mottle Virus (ToMoV). Using biolistic inoculation, the ToMoV-based VIGS vector was shown to be effective in *S. peruvianum* by silencing the magnesium chelatase gene, resulting in leaf bleaching. VIGS with the ToMoV_*SCE*I construct resulted in ~61% silencing of *SCE*I in leaves of *S. peruvianum* as determined by quantitative RT-PCR. The *SCE*I-silenced plants showed unilateral wilting (15 dpi) and subsequent death (20 dpi) of the entire plant after *Cmm* inoculation, whereas the empty vector-treated plants only showed wilting in the *Cmm*-inoculated leaf. The *SCE*I-silenced plants showed higher *Cmm* colonization and an average of 4.5 times more damaged tissue compared to the empty vector control plants. *SCE*I appears to play an important role in the innate immunity of *S. peruvianum* against *Cmm*, perhaps through the regulation of transcription factors, leading to expression of proteins involved in salicylic acid-dependent defense responses.

## Introduction

*Clavibacter michiganensis* subsp. *michiganensis* (*Cmm*) is a Gram-positive plant bacterial pathogen belonging to the order Actinomycetales in family Microbacteraceae (Gartemann et al., 2008). It is the causal agent of bacterial wilt and canker of tomato *(Solanum lycopersicum)*, which occurs worldwide (Eichenlaub and Gartemann, 2011). Bacterial wilt and canker has been reported to cause losses of tomato production as high as 84% in the U.S.A. and Canada (Strider, 1969; Gleason et al., 1993). *Cmm* can infect through wounds entering the xylem vessels producing enzymes, such as, endocellulases, polygalacturonases, pectin methylesterases, xylanases, serine proteases, and endo-1,4-glycosidases, that render the xylem non-functional (Carlton et al., 1998; Jahr et al., 1999, 2000). The symptoms begin as a unilateral wilting of leaves, followed by a generalized wilting of all the leaves and the cankers development on the stem. Cankers on young plants are particularly damaging as they can result in plant death. *Cmm* also infects fruit, which results in necrotic spots called bird's eyes (Gartemann et al., 2003).

Control of bacterial canker is difficult. Antibiotics are effective but can lead to selection of resistant bacterial populations (Strider, 1969; Gartemann et al., 2003). Cultural control can be achieved using certified disease-free seeds and strict hygienic measures, such as the removal and destruction of infected plants and compost biofumigation with compost (Gartemann et al., 2003). However, resistance is a desirable trait. Although there are no *Cmm*-resistant tomato cultivars commercially available, resistance has been identified in several wild tomato species, such as *S. pimpinelifolium, S. peruvianum*, and *S. habrochaites* (van Heusden et al., 1999; Kabelka et al., 2002; Coaker and Francis, 2004).

Using cDNA-AFLP analysis, a number of genes were found that were up-regulated in *Cmm* resistant *S. peruvianum* in comparison to *Cmm* susceptible *S. lycopersicum* plants following inoculation with *Cmm* (Lara-Ávila et al., 2012). One of those genes was the SUMO E2 conjugating enzyme SCE1 (SCEI), which encodes an enzyme involved in protein modification through sumoylation, which is a post-translational modification that covalently conjugates the small ubiquitin-like modifier (SUMO) protein to lysines on target proteins. Proteins labeled with SUMO are then modified by the addition of small chemical groups, such as sugars and lipids or by the covalent attachment of other proteins. Sumoylation is a multistep process mediated by E1 (SUMO activating enzyme), then E2 SCEI (SUMO conjugating enzyme) and finally E3 (SUMO ligase) (Berndsen and Wolberger, 2014).

SCEI has been shown to increase during plant-pathogen interactions. Expression of *SCE*I increased during *Pseudomonas syringae* pv. *tomato* (*Pst*) infection of tomato plants, along with several hormones, such as salicylic acid (SA) and jasmonic acid (JA), which are key signaling molecules in innate immunity (Miura and Hasegawa, 2010; van den Burg et al., 2010; Park et al., 2011). Overexpression of *SCE*I in *Arabidopsis* did not have obvious effects on plant development but increased expression of abscisic acid (ABA)-responsive genes following ABA treatment. ABA is also linked to innate immunity through its positive effects on callose deposition (Lois et al., 2003). In addition, SCEI promotes SUMO conjugation, which affects innate immunity due to its involvement in SA-dependent resistance to bacterial pathogens (van den Burg et al., 2010; Park et al., 2011). Also some pathogen effectors, which suppress innate immunity, such as AvrBst of *Xanthomonas campestris* pv. *vesicatoria*, act as desumoylation enzymes, indicating a link between sumoylation and innate immunity (Xia, 2004).

**Figure 1:**
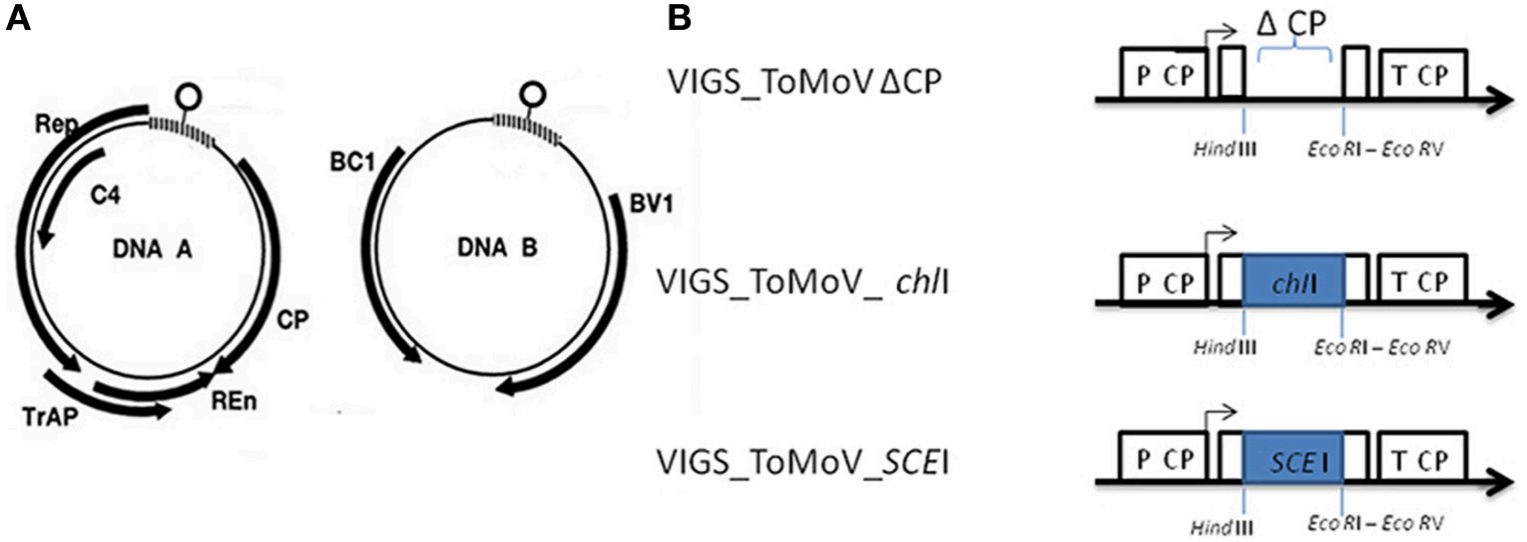
**ToMoV silencing vector**. **(A)** Diagram of the five genes of ToMoV component A: replication-associated protein (*Rep*), transcription activator protein (*Trap*), replication enhancer (*Ren*), coat protein (*CP*), and the two genes of ToMoV component B: movement proteins (*BC*1 and *BV*1) (Figure 1A modified from Gutierrez, 2002). **(B)** Organization of the modified ToMoV vector used in VIGS of *ChI*I and *SCE*I genes. The capsid's promoter is shown as P CP. The capsid protein gene (*CP*) was deleted by reverse PCR to generate Δ *CP*, and portions of *ChI*I and *SCE*I were cloned into this region with *EcoR*I and *Hind*III.

One approach to evaluate the role of *SCE*I and sumoylation in plant disease resistance is to down-regulate its expression. Virus-induced gene silencing (VIGS) involves the production of dsRNA that directs DICER complexes for degradation of desired sequences resulting in effective plant gene silencing (Liu et al., 2002; Robertson, 2004; Galun, 2005; Cai et al., 2007). In this study, a novel VIGS vector was developed for wild tomato species by modifying a Tomato Mottle Virus (ToMoV), which is a Begomovirus that infects many wild tomato species, such as *S. peruvianum* (Polston et al., 1993) and *S. habrochaites*, and does not cause a drastic phenotypic effect on either of those species (Esparza-Araiza et al., unpublished). ToMoV contains two single-stranded circular DNAs (DNA A and B). DNA A has the genes *Rep* for virus replication, *C4* for infectivity and suppression of posttranscriptional gene silencing, *Trap* for transactivation of *BC*1 and *BV*1, *Ren* for increased multiplication efficiency and *CP* for viral capsid. DNA B has the genes *BC*1 and *BV*1 for viral movement (Jeske, 2009) (Figure 1).

The goals of this study were to determine the effectiveness of a modified ToMoV vector for VIGS in *S. peruvianum* and to use the modified ToMoV as a vector for VIGS of *SCE*I. The effectiveness of a modified ToMoV VIGS vector was demonstrated by silencing a magnesium chelatase gene (*Chl*I) since its silencing causes leaf bleaching due to the suppression of the magnesium chelatase complex which is required for chlorophyll production (Cai et al., 2007). A portion of the *SCE*I gene from *S. peruvianum* was cloned into the modified ToMoV vector, introduced into *S. peruvianum* by particle bombardment, and then the *SCE*I-silenced plants were evaluated for their degree of resistance to *Cmm*.

## Materials and methods

### Virus, bacterial strain, plant material, inoculum, and challenge procedure

The sequence of ToMoV was obtained from a strain isolated from tomato leaves in Florida, USA in 2003 (GenBank accession nos. NC_00193 and NC_001939). Strain 1387 of *Cmm* was isolated from a commercial tomato field near Queretaro, Mexico in 2012 by J. P. Lara-Avila, Universidad Autonoma de San Luis Potosi, MX. *Cmm* was grown in 802 medium broth (polypeptone 1 g L^−1^, yeast extract 2 g L^−1^, MgSO_4_·7H_2_O 0.92 g L^−1^) at 28°C for 48 h shaking at 190 rpm. The bacterial suspension was diluted to 5 × 10^7^ CFU/mL^−1^ (*A*_620_ = 0.2), and 0.5 mL was injected with an insulin syringe into the lower side of the first true leaves of 2 month-old of *S. peruvianum*. Seeds of *S. peruvianum* accession LA2172 were obtained from Tomato Genetics Resources Center, Davis, California, and seeds of *S. lycopersicum* cv. Ailsa Craig were obtained from the University of Nottingham, UK. Plants were grown in a commercial soil mixture substrate (Sunshine Mix #6, Sun Grow Horticulture, Vancouver, BC, CA), in individual pots in growth chambers at 25°C with a 16 h/8 h light/dark regime and then transferred to a greenhouse.

### Silencing vector design and cloning of *ChI*I and *ScE*I genes

DNA of ToMoV A and B components were extracted from tomato leaves by R. F. Rivera-Bustamante, CINVESTAV, IPN, Mexico. The complete DNA of the ToMoV A and B components were cloned into pBluescript [pBS II SK (+∕−)] (Stratagene, La Jolla, CA, USA) using *Apa*I digestion. These constructs were named pBS_ToMoV A and pBSToMoV B. The pBS_ToMoV A construct was modified by removing a 51-bp fragment between a *Xho*I (668 bp position) and *Bam*HI sites (719 bp position), as it would have interfered with the use of the multiple cloning site of pBS II SK (+∕−). The cloned ToMoV A capsid gene was then modified by removing a 657 bp region using PCR with the forward primer 5′-CTGAATTCAAGCTTTGCACT CATGCGTCTAACCCTG-3′ and reverse primer 5′-TCGAATTCGATATCCC ATGGCAAATCACGCTTAGGC-3′ that flanked the capsid gene. The forward primer was designed with *EcoR*I and *Hind*III restriction sites and the reverse primer was designed with *EcoR*I and *EcoR*V restriction sites. The PCR conditions were 7 min at 95°C, followed by 35 cycles of 1 min at 94°C, 1 min at 55°C, 5.5 min at 72°C, and 10 min at 72°C. The PCR product was purified, digested with *EcoR*I enzyme and ligated subsequently to pBS II SK(+/–) (Stratagene, La Jolla, CA, USA). The ligation mix was used to transform *E. coli* Top 10 strain (Invitrogen, Carlsbad, CA, USA). This construct was named ToMoVΔCP.

For VIGS of *ChI*I, a 249-bp portion of the chelatase gene was amplified using genomic DNA of *S. peruvianum* LA2172 with the forward primer 5′-CTGCAGGAATTCCTC CAGAGCCAAATCACCTC-3′ and reverse primer 5′-AAGCTTAG ATTCCAACGGATCCTTCC- 3′. The forward and reverse primers were designed with *EcoR*I and *Hind*III restriction sites, respectively. These primers were designed based on the *S. lycopersicum*'s chelatase sequence (XM_004248092.1). The PCR conditions were 5 min at 94°C, 50 s at 94°C, 40 s at 60°C, 50 s at 72°C for 35 cycles, and then 5 min at 72°C. The PCR product was purified and ligated to ToMoVΔCP-VIGS following digestion with *EcoR*I and *Hind*III. This plasmid was named ToMoV_*ChI*I.

For VIGS of *SCE*I, genomic DNA of *S. peruvianum* LA2172 was used as a template in PCR with forward primer 5′-CTCGAATTCTCCTCAATGAAG ACAGTGGTTGG-3′and reverse primer 5′-ATAAAGCTTCA CCCTCTTTCGGTACTCCA-3′, containing *EcoR*I and *Hind*III restriction sites, respectively. Theses primers were designed based on the sequence (emb|CAE45567.1) Lara-Ávila et al. (2012). The 169-bp PCR product and the ToMoVΔCP-VIGS vector were digested with *EcoR*I and *Hind*III and then ligated. This plasmid was named ToMoV_*SCE*I. The identity of all constructs was confirmed by restriction analysis with *EcoR*I and *Hind*III and sequencing.

### Plant inoculation with the ToMoV VIGS vector

ToMoV virus A and B components were mixed in a 1:1 ratio (1 μg of component A: ToMoVΔCP, ToMoV_*ChI*I or ToMoV_*SCE*I and 1 μg of component B: ToMoV B) after their digestion from the pBS II SK vector with *Apa*I. Twenty-two day-old plants were treated with the virus mixture using the Biolistic® PDS-1000He gun (Bio Rad, Hercules, CA, USA) at low pressure (Carrillo-Tripp et al., 2006). The plants were then maintained for 60 days post-treatment (dpt) in a greenhouse at 25–30°C before inoculation with *Cmm*. This time period was chosen because ToMoV_*ChI*I inoculated plants showed bleaching of all leaves by 60 days, which indicated that silencing had occurred. By 15 days post inoculation (dpi) with *Cmm*, symptoms of leaf wilting and necrosis were observed and recorded by scanning excised damaged leaves on a flat-bed scanner. A tif file was created and the number of pixels of damaged tissue was quantified by Scion Image (Scion Corporation, Frederick, MD, USA) (Wijekoon et al., 2008). Statistical analysis was based on *T*-test with unpaired data with Graph Pad Prism® V.5 (GraphPad, San Diego, CA, USA), and a statistically significant result was considered to be *P* < 0.01.

### RNA isolation and quantitative RT-PCR analysis

In order to quantify *SCE*I mRNA from silenced and empty vector control plants, total RNA was isolated using Trizol (Invitrogen, Carlsbad, CA, USA) from 40 dpt *S. peruvianum* (62 days old plants) inoculated either with ToMoVΔCP or ToMoV_*SCE*I. After treatment with DNAse I (Invitrogen, Carlsbad, CA, USA), RNA was quantified by a NanoDrop ND-1000 UV-Vis spectrophotometer (NanoDrop Technologies Inc., Wilmington, DE, USA) according to the manufacturer's instructions. cDNA synthesis and quantitative real time PCR analysis were performed using the iScript™ One-Step RT-PCR kit with SYBR® Green (Bio-Rad Laboratories, Hercules, CA, USA). The 20 μL reactions contained 100 ng of total RNA, 12.5 μL of 2x SYBR Green RT-PCR reaction mix, 200 nM of each primer listed below and 1 μL of iScript MMlV reverse transcriptase. Quantification was based on a cycle threshold value with expression level of *SCE*1 gene normalized to actin gene (Accession no. FJ532351). The *SCE*I forward primer 5′-TTGCTAAGCCGGA GACACTT-3′ and reverse primer 5′-ACACTTTGGC GGTTTACTCG-3′ were designed outside the targeted region for silencing. For actin, the forward primer was 5′-CCTCACCGAGAGAGGTTACA TGT-3′ and reverse primer was 5′-CATGTCGCGGACAATTTCC3′. The RT-PCR conditions were 10 min at 50°C (cDNA synthesis), 5 min at 95°C (iScript MMLV reverse transcriptase inactivation), followed by 40 PCR cycles of 10 s at 95°C and 30 s at 60°C. Melting curves were performed by 80 cycles of 1 min at 95°C, 1 min at 55°C, and 10 s at 55°C increasing the temperature by 0.5°C per cycle of 10 s each. Absence of contaminating genomic DNA was confirmed by PCR of RNA samples without cDNA synthesis. PCRs were performed on an Applied Biosystems 7500 Fast Real-Time Real-Time-PCR system version 2.0, and the data was analyzed with the Applied Biosystems 7500 software V.2.0. Three biological replicates were analyzed with three technical replicates per biological replicate. Statistical analysis was based on *T*-tests with unpaired data with Graph Pad Prism® V.5 (GraphPad, San Diego, CA, USA), and a statistically significant result was considered to be *P* < 0.01.

### *Cmm* DNA detection in inoculated plant tissue by PCR

Total DNA was isolated from *S. peruvianum* plants at 10 and 20 dpi with ToMoVΔCP or ToMoV_*SCE*I based on a modified protocol of Dellaporta et al. (1983), and then quantified with a NanoDrop ND-1000 UV-Vis Spectrophotometer (NanoDrop Technologies Inc., Wilmington, DE, USA). A 233-bp of the endo-1,4-beta-glucosidase gene, *Cel*-A (HQ636581; Lara-Ávila et al., unpublished) was amplified using 100 ng of the DNA as template and forward primer 5′-ATCAAGCAGATGG GGTTCAC-3′ and reverse primer 5′-TCCGGATACTGCGA TGTGTA-3′. The PCR conditions were 5 min at 94°C, and then 50 s at 94°C, 40 s at 60°C, 50 s at 72°C for 35 cycles followed by 5 min at 72°C.

### *Cmm* DNA quantification in inoculated plant tissue by quantitative PCR

Total DNA was isolated and quantified as above from *S. peruvianum* plants inoculated either with ToMoVΔCP or ToMoV_*SCE*I. For a 20 μL reaction, 100 ng of the DNA was added as template to 10 μL SYBR Green RT-PCR master mix (Applied Biosystems, Carlsbad, CA, USA) and 200 nM each of forward primer 5′-GAGCCAAGCCAC TGATCTTC-3′ and reverse primer 5′-CGTTCT CGTAGAGGCGGTAG-3′ to generate a 219 bp portion of the tomatinase, endo-1,4-beta-glycosidase constitutive gene, *Tom*A, of *Cmm* (AF393183.1; Flügel et al., 2012), RT-PCR, melting curve, quantification and data analysis were performed as per *SCE*I described previously. A standard curve was created based on the concentrations of a cloned version of *Tom*A in the pGEM-T Easy vector (Promega, Madison, WI, USA) using 2.86 × 10^2^ to 2.86 × 10^8^ copies/ng. The correlation coefficient between the cycle threshold value and the concentration of the cloned *Tom*A was of 0.999. Statistical analysis was based on a correlation test with unpaired data with Graph Pad Prism® V.5 (GraphPad, San Diego, CA, USA), and a statistically significant result was considered to be *P* < 0.01.

### Scanning electron microscopy

One-half centimeter stem samples were excised and fixed with glutaraldehyde 3% in Sörensen buffer (100 mM sodium phosphate pH 7.4). After washing three times in buffer, the samples were immersed in 1% osmium tetraoxide (OsO4) in Sörensen buffer for 2 h, washed three times with Sörensen buffer, and then dehydrated with ethanol 30, 50, 70, 90, 95% and absolute ethanol for 15 min each, and then incubated two additional times in absolute ethanol for 15 min. Critical point dried were done in a Tousimis Samdri-PVT-3D (Tousimis Research, Rockville, MA), mounted and gold coated sputter in Cressington model 108auto (Cressington Scientific Instruments, Watford, UK) and examined in a FEI model Quanta 200 SEM (FEI, Brno, Czech Republic).

## Results

### Silencing *ChI*I using the ToMoV VIGS vector in *S. peruvianum*

**Figure 2:**
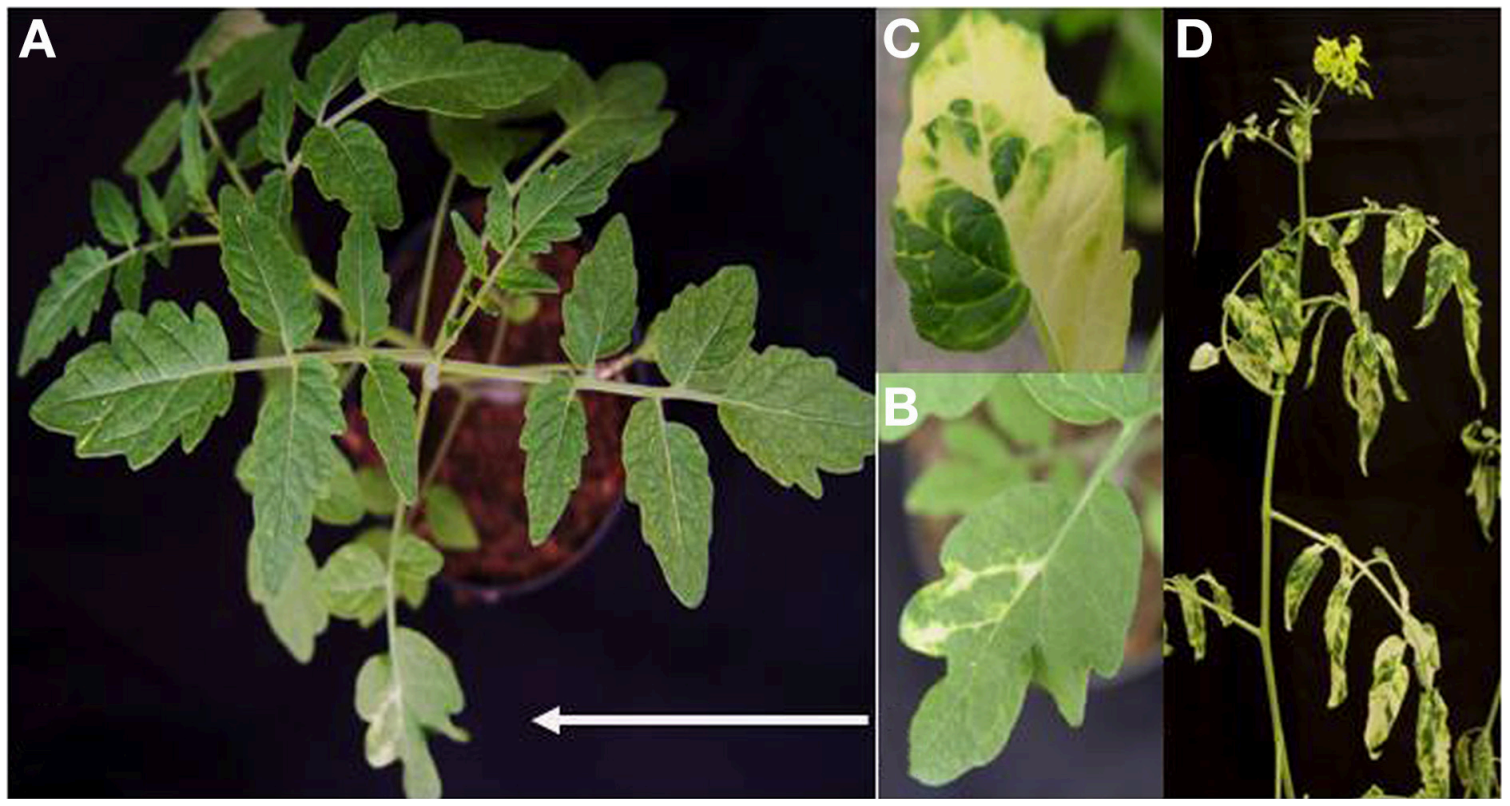
**Phenotype of silencing of ***ChI***I gene in ***S. peruvianum***. (A)** Phenotype of leaf bleaching at 10 dpi. **(B)** Close up of a bleached leaf at 10 dpi. **(C)** Close up of a bleached leaf 40 dpi. **(D)** Bleaching phenotype at 40 dpi.

In order to develop a VIGS vector based on ToMoV, the coat protein contained in component A of ToMoV was removed, and a cloning site was added at the same location (Figure 1). A portion of the magnesium chelatase gene from *S. peruvianum* was cloned into the ToMoV VIGS vector (ToMoV_*ChI*I) and introduced by bombardment into 22 day old *S. peruvianum*. Typical leaf bleaching indicating silencing of *Chl*I first appeared at 10 dpi and spread from the bombarded leaf until the whole plant was showing patchy bleaching at 40 dpi (Figure 2). Control plants inoculated with an empty ToMoV vector (ToMoVΔCP) did not show any bleaching symptoms. Similar results were obtained when *Chl*I was silenced in *S. habrochaites, S. lycopersicum* cv. Micro Tom, *S. lycopersicum* cv. Ailsa Craig, and *Nicotiana benthamiana* (data not shown).

### Silencing of *SCE*I using the ToMoV VIGS vector in *S. peruvianum*

**Figure 3:**
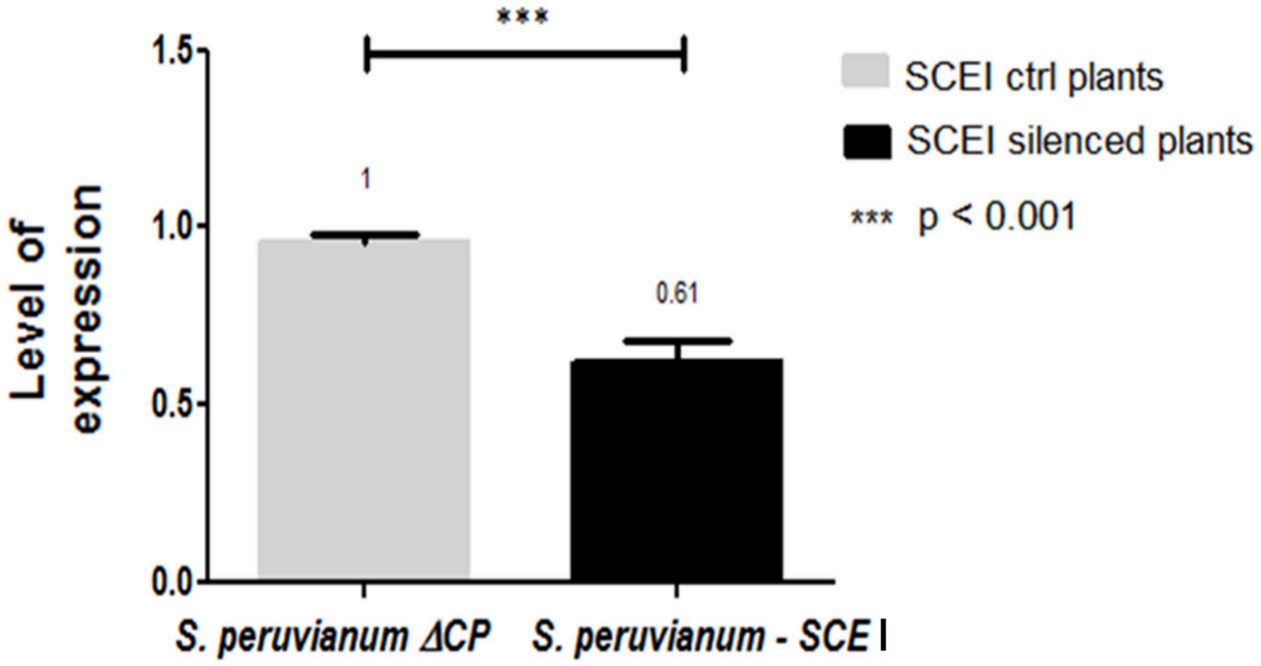
**Expression level of the ***SCE***I gene in ***S. peruvianum*** plants after silencing with ToMoV – VIGS vector**. Real Time one step RT-PCR amplifications were performed using 100 ng of total RNA with the iScript™ One-Step RT-PCR kit using SYBER® Green. Quantification was based on the cycle threshold value with the expression level of *SCE*I normalized to that of *S. peruvianum* actin.

**Figure 4:**
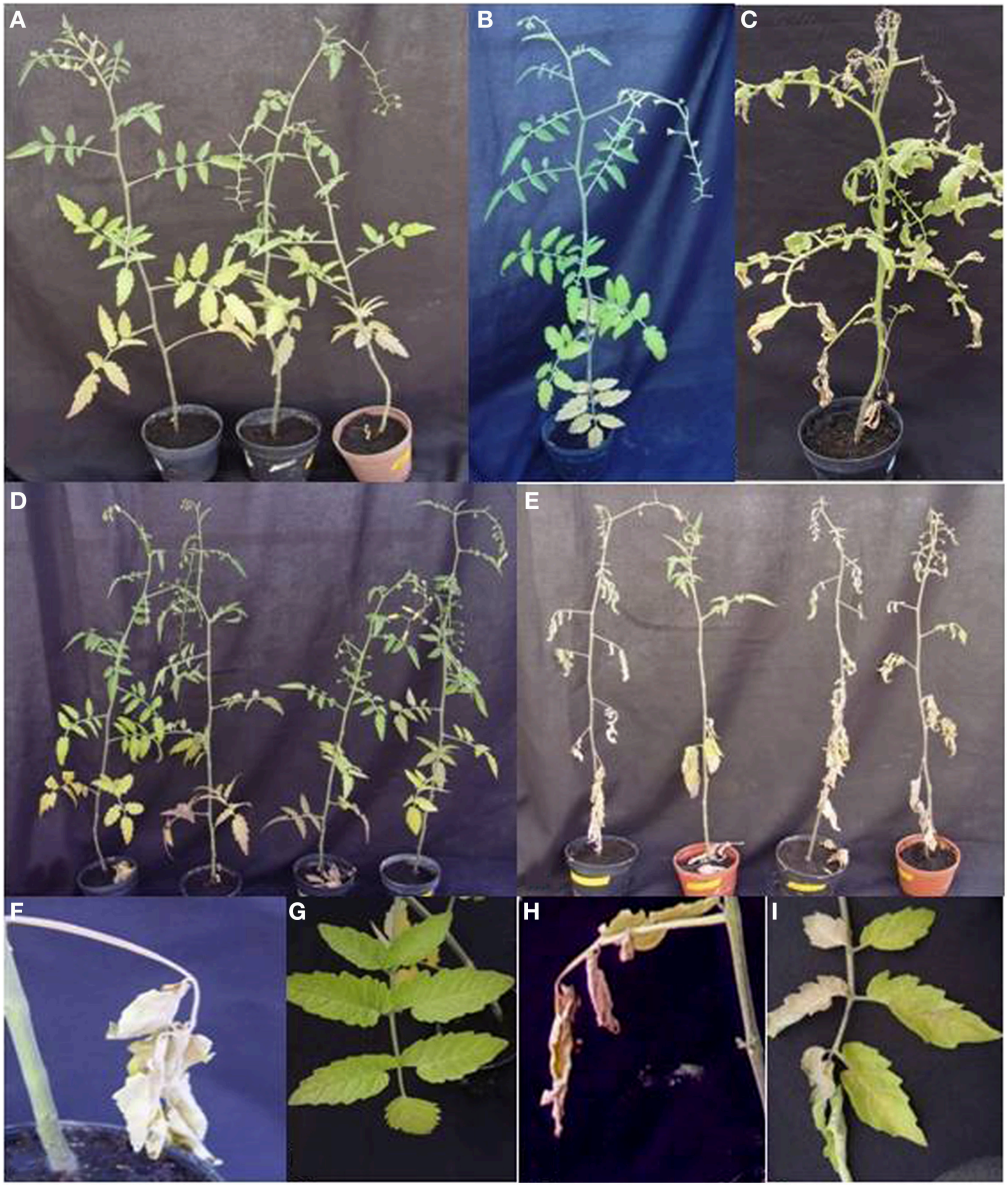
**Phenotypes of ***S. peruvianum*** and ***S. lycopersicum*** plants inoculated with ToMoV VIGS vector prior and 20 days post-infection with ***Cmm*****. **(A)** Phenotype of *SCE*I-silenced *S. peruvianum* plant without *Cmm* inoculation. **(B)** non-silenced *S. peruvianum* plant without *Cmm* inoculation (negative control). **(C)** non-silenced *S. lycopersicum* plant inoculated with *Cmm*. **(D)** empty vector-treated *S. peruvianum* plants inoculated with *Cmm*. **(E)** *SCE*I-silenced *S. peruvianum* plant inoculated with *Cmm*. **(F)** Close up of the *Cmm* infection site on the *S. peruvianum* leaf for an empty vector-treated plant. **(G)** Close up of the *S. peruvianum* leaf located above the *Cmm* infection site for the same empty vector-treated plant, **(H)** Close up of the *Cmm* infection site on the *S. peruvianum* leaf for a *SCE*I-silenced plant. **(I)** Close up of the *S. peruvianum* leaf located above the *Cmm* infection site for the same *SCE*I-silenced plant showing unilateral wilting.

To silence *SCE*I, a 269 bp portion of the gene from *S. peruvianum* was amplified and cloned into the ToMoV VIGS vector (Figure 1B). Expression of *SCE*I at 40 dpi for *S. peruvianum* inoculated with ToMoV_*SCE*I was 0.61 of the value of plants inoculated with the empty vector (Figure 3) (*t*-test, *p* < 0.001) indicating silencing. The plant morphology and flowers of *S. peruvianum* inoculated with ToMoV_*SCE*I was identical to that of empty vector control plants (Figures 4A,B). Thus, silencing of *SCE*I did not have any apparent effect on the healthy plant phenotype under the conditions used in these experiments.

### *SCE*I silencing is associated with disease susceptibility and increased *Cmm* growth in *S. peruvianum* plants

**Figure 5:**
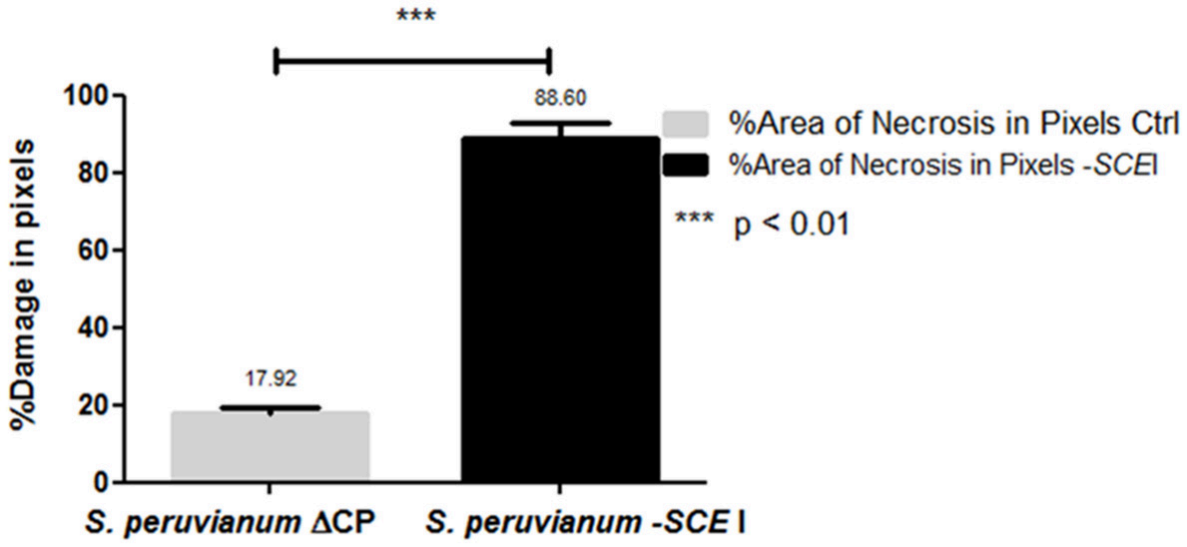
**Damaged leaf tissues in empty vector-treated or SCEI-silenced ***S. peruvianum*** plants at 20 dpi with ***Cmm*****.

*Cmm*-inoculated *S. peruvianum* silenced for *SCE*I first showed unilateral wilting of leaves at 15 dpi (Figures 4H,I), and the wilting spread to all leaves at 20 dpi resulting in plant death (Figure 4E). Plants inoculated with the empty vector showed necrosis only in the leaf that was inoculated with *Cmm* (Figure 4F), and the rest of the plant appeared healthy without any symptoms typical of *Cmm* infection for up to 20 dpi (Figures 4D,G). In contrast, the susceptible cultivar, *S. lycopersicum* cv. Ailsa Craig, showed unilateral wilting of leaves at 15 dpi, and the wilting spread to all leaves at 20 dpi (Figure 4C). The amount of damaged tissue in *S. peruvianum* plants due to *Cmm* infection varied between 12.57% to almost 25% in plants inoculated with ToMoVΔCP, whereas it was between 70.53 and 100% in plants inoculated with ToMoV_*SCE*I (*t*-test, *p* < 0.01) (Figure 5). Thus, the percentage of necrosis was approximately five times lower in the empty vector control than in the *SCE*I-silenced plants.

**Figure 6:**
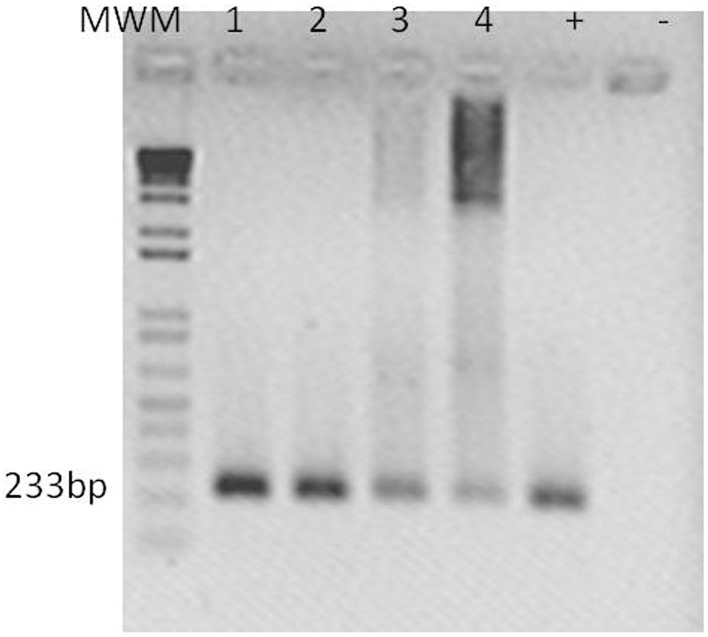
*****Cmm*** detection in ***S. peruvianum*** tissues by PCR**. An amplicon of 233 bp was obtained using *Cel-*A primers por *Cmm* detection at 10 and 20 dpi. Lanes show PCR products shown with template of: (1) leaf tissue of *SCE*I-silenced *S. peruvianum* at 10 dpi, (2) leaf tissue of *SCE*I-silenced *S. peruvianum* at 20 dpi, (3) leaf tissue of empty vector treated *S. peruvianum* at 10 dpi, (4) leaf tissue of empty vector treated *S. peruvianum* at 20 dpi. Plasmid of pGEM T-Easy with Cel-A fragment. (-) Negative control. 100 ng of DNA were taken for each reaction.

**Figure 7:**
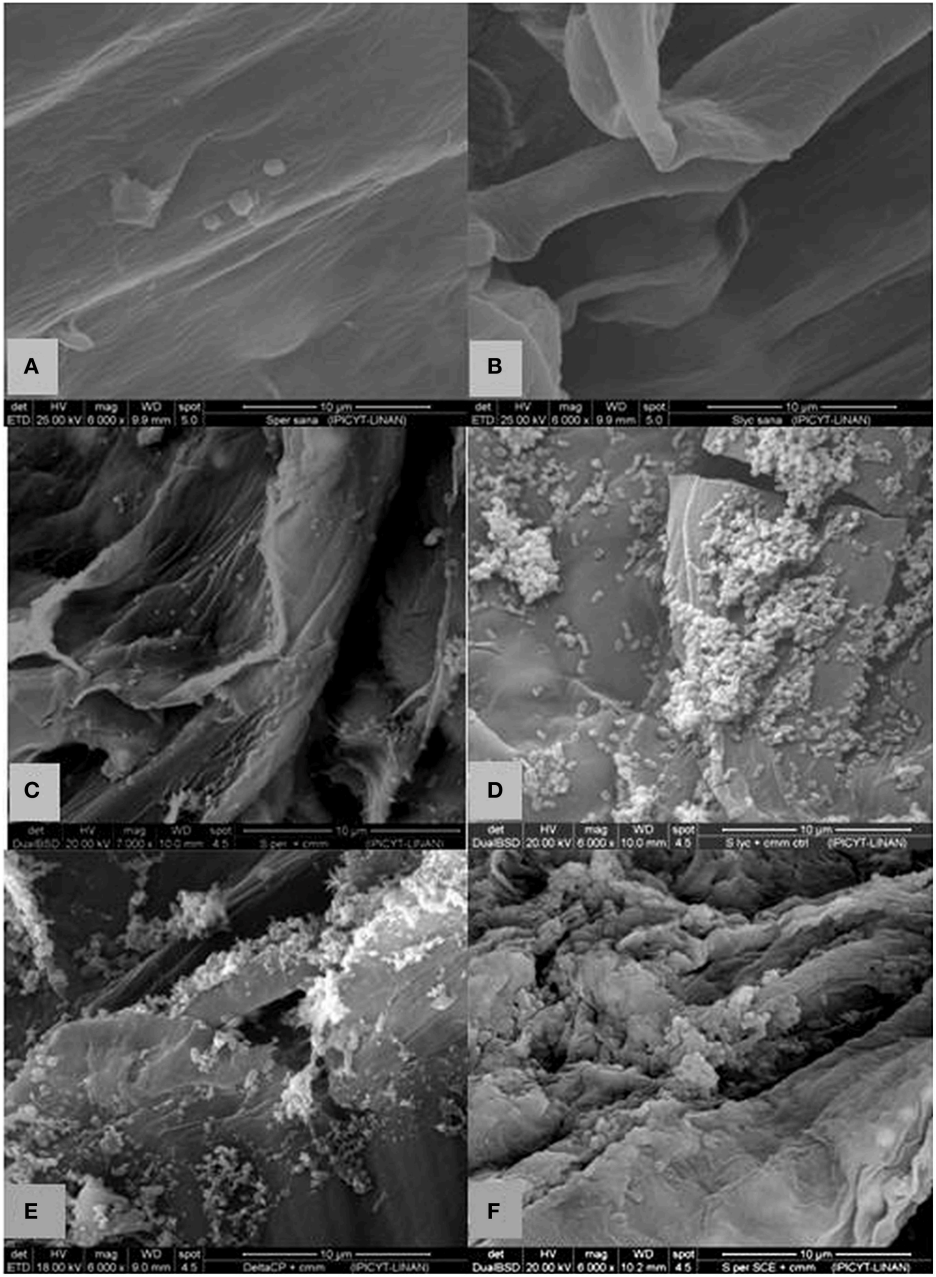
**Scanning electron microscopy of longitudinal cut of a stem of non-treated, empty vector and ***SCE***I-silenced ***Solanum*** plants with and without ***Cmm*** challenge**. **(A)** *S. peruvianum* without inoculation of *Cmm* showing no bacteria or tissue damage **(B)** *S. lycopersicum* without inoculation of *Cmm* showing no bacteria or tissue damage **(C)** non-VIGS treated *S. peruvianum* at 10 dpi with *Cmm* showing some bacterial but limited parenchymal tissue damage. **(D)** *S. lycopersicum* at 10 dpi with *Cmm* revealing more bacterial structures with greater parenchymal tissue damage. **(E)** empty-vector treated *S. peruvianum* at 10 dpi with *Cmm* with some bacterial structures and limited parenchymal tissue damage. **(F)** *SCE*I-silenced *S. peruvianum* at 10 dpi with *Cmm* revealing considerable bacterial structures and parenchymal tissue damage. Magnification 6000x, scale bar ~10 μm.

The presence of *Cmm* in inoculated plants was confirmed by PCR using specific primers for *Cmm Cel*-A using DNA obtained from ~3 cm above the inoculation site (Figure 6). SEM of non-infected *S. peruvianum* and *S. lycopersicum* plants showed that the parenchymal tissues appeared intact, healthy and without bacterial cells (Figures 7A,B). However, in *S. peruvianum* inoculated with ToMoVΔCP and challenged with *Cmm*, some bacteria were observed in the parenchymal tissue with little to no parenchymal tissue damage (Figures 7C,E). In contrast, *S. peruvianum* inoculated with ToMoV_*SCE*I and challenged with *Cmm* had more bacteria in the parenchymal tissue and more parenchymal tissue damage when compared with empty vector-inoculated control plants (Figure 7F). However, the number of bacteria and the level of parenchymal tissue damage were less than in the susceptible *S. lycopersicum* cv. Ailsa Craig, where the highest number of *Cmm* and the most parenchymal tissue damage was visible (Figure 7D). The quantity of bacteria observed by SEM correlated with the visible symptoms observed in empty vector inoculated *S. peruvianum, SCE*I silenced *S. peruvianum*, and susceptible *S. lycopersicum*, cv. Alisa Craig.

Real time PCR of *Tom*A of *Cmm* was performed to quantify the *Cmm* population. At 10 dpi, the *Cmm* population reached 3.77 × 10^8^ CFU per g of stem in *SCE*I-silenced *S. peruvianum*, whereas the population only reached 6.80 × 10^7^ CFU per g of stem in the empty vector control *S. peruvianum* (*t*-test, *p* < 0.05) (Table 1). At 20 dpi, the population in the *S. peruvianum SCE*I-silenced plants increased to 1.23 × 10^9^ CFU per g of stem, which was significantly higher than the 1.11 × 10^8^ CFU per g of stem in the empty vector silenced plants (*t*-test, *p* < 0.05). Thus, *Cmm* populations were about 5–10 times higher due to *SCE*I silencing in *S. peruvianum*. At 10 dpi, the susceptible *S. lycopersicum* cv. Ailsa Craig had a significantly higher population of 4.63 × 10^8^ CFU per g of stem, and at 20 dpi, the *Cmm* population reached 3.00 × 10^10^ CFU per g of stem, which as significantly higher than in *S. peruvianum* for both the *SCE*I-silence and empty vector control. These population differences correlated well with the amount of necrosis observed in the different plants.

**Table 1:** *****Cmm*** populations estimated by qRT-PCR of the constitutive ***Cmm*** TomA gene in inoculated stems of ***S. peruvianum*** inoculated with ToMoVΔCP or ToMoV_***SCE***I or ***S. lycopersicum*** cv**.

**Samples**	**Days post inoculation**	**CFU/g ± SD**
*S. peruvianum*Δcp	10	6.80 × 10^7^ ± 4.8 × 10^6^
*S. peruvianum*Δcp	20	1.11 × 10^8^ ± 9.4 × 10^6^
*S. peruvianum*-SCEI	10	3.77 × 10^8^ ± 4.7 × 10^6^
*S. peruvianum*-SCEI	20	1.23 × 10^9^ ± 2.0 × 10^7^
*S. lycopersicum*	10	4.63 × 10^8^ ± 3.4 × 10^7^
*S. lycopersicum*	20	3.00 × 10^10^ ± 6.2 × 10^8^

*Ailsa Craig not inoculated with the VIGS vector*.

## Discussion

VIGS is a tool that has been used successfully for the analysis of gene function without the time-consuming need to generate mutants or transgenic plants (Liu et al., 2002; Burch-Smith et al., 2004). For *S. lycopersicum*, there have been several viruses used for VIGS, such as Potato Virus X (Giliberto et al., 2005) and DNA1 component of tobacco curly shoot virus (TbCSV) (Huang et al., 2009), but the most commonly used VIGS vector has been based on Tobacco Rattle Virus (TRV) (Liu et al., 2002). However, there is one report of VIGS with the wild tomato, *S. peruvianum*, using TRV (Senthil-Kumar et al., 2007). In this work, a new VIGS vector based in ToMoV was developed, which showed silencing of genes in *S. peruvianum* and other *Solanum* species. The vector produced no or barely any viral symptoms in those *Solanum* species, and was an effective VIGS vector in *S. peruvianum* based on silencing a chelatase gene that blocks chlorophyll production resulted in large areas of yellowed and bleached tissues, similar to the phenotype reported in other plant species with chelatase silencing (Ekengren et al., 2003; Burch-Smith et al., 2004; Cai et al., 2007). An advantage of the ToMoV VIGS vector over TRV vectors is that silencing of genes with ToMoV was effective in *S. peruvianum* and other *Solanum* species at temperatures ranging from 22 to 30°C (data not shown). However, for TRV, slightly higher or lower temperatures from 21°C can greatly affect VIGS in tomato (Cai et al., 2007). Based on these results, the ToMoV VIGS vector was used to silence our gene of interest in *S. peruvianum* to determine if it may be involved in plant defenses against *Cmm*.

*SCE*I was selected for VIGS because Lara-Ávila et al. (2012) demonstrated that expression of this gene was highly up-regulated in the early stage of *Cmm* infection in *S. peruvianum*, which was followed by a decline to basal levels later in the infection, suggesting a role in the early defense response. In contrast, *S. lycopersicum* showed no significant change in *SCE*I expression levels following *Cmm* infection. All *S. lycopersicum* genotypes are relatively susceptible to *Cmm*, but *S. peruvianum* has a high level of resistance to this bacterial pathogen (van Ooijen et al., 1994). Inoculation of *S. peruvianum*, either on its own or with the empty ToMoV vector, only produced a rapid localized cell death at the inoculation site, whereas susceptible *S. lycopersium* cv. Ailsa Craig and *SCE*I-silenced *S. peruvianum* showed a necrotic phenotype of the leaves ranging from 70 to 100%. In addition, the population size of *Cmm* in *S. peruvianum* without treatment or treated with the empty ToMoV vector was much lower than in the *SCE*I-silenced *S. peruvianum* or *S. lycopersicum* cv. Ailsa Craig, whose populations reached levels similar to those of *Cmm* reported on susceptible *S. lycopersicum* cv. Moneymaker and *S. lycopersicum* cv. Jet Star by Sen et al. (2013) and Carlton et al. (1998), respectively.

Plant innate immunity is a multi-step process beginning with pathogen recognition. One type of innate immunity is based on recognition of Pathogen-Associated Molecular Patterns (PAMPs), which are invariant epitopes within molecules that are fundamental to the pathogens fitness, widely distributed among different microorganisms (Schwessinger and Zipfel, 2008). Another type of innate immunity is based on recognition of effectors, which generally are secreted by pathogens to manipulate or reprogram host defenses (Zipfel, 2008). The two types of recognition result in PAMP triggered immunity (PTI) and effector triggered immunity (ETI), which is usually stronger and longer than PTI and is often associated with the hypersensitive response (HR) at the infection site (Tsuda and Katagiri, 2010; Meng and Zhang, 2013). Both ETI and PTI initiate common signaling pathways differing in length and amplitude, such as an oxidative burst, activation of transcription factors, and MAP kinases and the production of plant hormones (Chisholm et al., 2006). Eventually, the plant responds through the expression of defense genes resulting in the production of various PR proteins and antimicrobial compounds and structures that limit pathogen spread and reproduction (Kaup et al., 2005; Saracco et al., 2007; Miura and Hasegawa, 2010; van den Burg et al., 2010; Balaji et al., 2011). The observation of rapid localized necrosis at the *Cmm* inoculation site in *S. peruvianum* indicates that the HR and ETI may be involved in *Cmm* resistance. In this study, SCEI-silenced plants did not have this localized cell death and clearly failed to restrict pathogen growth, similar to the susceptible *S. lycopersicum*, indicating that ETI could be compromised with diminished SCEI.

SUMOylation is a key process in plants as it provides post-translational modification of proteins involved in nuclear-cytosolic transport, transcriptional regulation, apoptosis, protein stability, response to stress and progression through the cell cycle and is controlled by SUMO pathway through regulation of transcription (Yang and Sharrocks, 2006; Enserink, 2015). The importance of SUMOylation in ETI can be inferred from studies of certain pathogen effectors. Hotson et al. (2003) found that the XopD effector of *Xanthomonas campestris* pathovar *vesicatoria* (*Xcv*) functions as cysteine protease with plant-specific SUMO substrate specificity. Roden et al. (2004) showed that the AvrXv4 effector of *Xcv* possesses SUMO isopeptidase activity, suggesting that SUMO conjugation system may be a key target for plant pathogen effectors. Therefore, some pathogen effectors act to hydrolyse SUMO-conjugated proteins to alter host cell signaling events, presumably for the pathogen's benefit (Hotson et al., 2003).

Several studies have suggested that SUMO plays an important role in pathogen defense responses (Lois et al., 2003; Saracco et al., 2007; van den Burg et al., 2010). One of the early defense genes with increased expression is *SCE*I (Pitzschke et al., 2009; van den Burg and Takken, 2010). Increased *SCE*I expression may be required in innate immunity because MAP kinase signaling and SUMOylation appear to converge to regulate the same targets that participate in signaling that controls defense gene expression (Yang and Sharrocks, 2006; Miller et al., 2010). Defense signaling involves a number of WRKY transcription factors, many of which have been identified as SUMOylation targets after phosphorylation by MAPKs. For example, WRKY3, WRKY4, WRKY6, WRKY33, WRKY72, and other WRKY transcription factors in *Arabidopsis* act as activators and/or repressor of defense gene expression and are also SUMO targets (Bethke et al., 2009; Popescu et al., 2009; Bhattarai et al., 2010; Miller et al., 2010; van den Burg and Takken, 2010). Therefore, silencing of the *S. peruvianum SCE*I gene by VIGS in this study may have made the SUMOylation mechanism on WRKYs or other MAP kinase targets non-functional, allowing for increased multiplication and development of disease symptoms by *Cmm*.

Increased levels of SCEI in plants, also occurs following abiotic stresses, such as salinity, drought, and cold. This was observed following salinity and drought stress in *Spartina alterniflora* (Karan and Subudhi, 2012). Lois et al. (2003) and Kurepa et al. (2003) also reported that high expression of *SCE* correlated with ABA mediated stress responses in different tissues of *Arabidopsis*, suggesting that sumoylation by SUMO1/2 played an early role in the plant stress response. ABA is well known for mediating plant stress responses to salinity, drought and cold (Karan and Subudhi, 2012). Thus, *SCE*I could also be involved in early stress responses following *Cmm* infection. One of the first responses to bacterial infections is a burst of ROS, and sumoylation is regulated by ROS (Zipfel and Robatzek, 2010; Park et al., 2011). On other hand, the overexpression of a SUMO gene in *Arabidopsis* resulted in activation of SA signaling following infections with *Pseudomonas syringae* pv *tomato* DC3000 and enhanced resistance to *Pst*DC3000 (Panstruga et al., 2009; van den Burg et al., 2010; Xiong and Wang, 2013). While SA and ROS are both signals in plant defense response, there is an antagonistic interaction between ROS and SA signaling (Xu and Brosché, 2014). Therefore, silencing *SCE*I in *S. peruvianum* could have made sumoylation non-functional, which could be affecting SA levels in response to ROS and thus affecting defense responses to *Cmm* infection. Baysal et al. (2003) and Balaji et al. (2008) found that SA is induced by acidbenzolar-S methyl (ASM) increased resistance in *S. lycopersicum* seeds. The best protection against *Cmm* was obtained when the ASM had been applied 3 days before the *Cmm* infection. If the SA response was not sufficiently activated, then *Cmm* may have been able to avoid SA-dependent defenses allowing it to reproduce to higher levels, spreading in the plant and eventually killing it.

Silencing SCEI could also have affected the defense response in *S. peruvianum* to *Cmm* by altering other factors in the plant, such as plant morphology. Null mutations of SCEI in *Arabidopsis* resulted in embryo lethality (Park et al., 2011), but in *Arabidopsis*, mutants with moderately reduced *SCE*I levels showed a normal phenotype suggesting that partially reduced levels of *SCE*I can be tolerated under non-stressed conditions (Saracco et al., 2007). Using VIGS to silence *SCE*I in *S. peruvianum* did not cause visible aberrant developmental effects, and thus this explanation appears unlikely for the loss of *Cmm* resistance in *S. peruvianum* following *SCE*I silencing.

While *S. lycopersicum* is susceptible to *Cmm*, this is not due to the lack of *SCE*I genes. The partial sequence of *SCE*I in *S. peruvianum* had 100% nucleotide identity with a *S. lycopersicum* sequence (Solyc02g093110) and lesser nucleotide identity with other *SCE*I genes from *S. lycopersicum* (Figure S1). Since sumoylation is involved in many processes other than pathogen resistance, *S. lycopersicum* must have a number of *SCE*I genes in order to survive. Although the coding region of the *SCE*I in *S. peruvianum* in this study and Solyc02g093110 may be identical, the results of Lara-Ávila et al. (2012) show that they are regulated very differently following *Cmm* inoculation. It is unknown at which stage that innate immunity to *Cmm* differs between *S. peruvianum* and *S. lycopersicum*, but *SCE*I regulation is a possibility. If so, then transgenic *S. lycopersicum* with Solyc02g093110 regulated by the promoter region of the *SCE*I gene from *S. peruvianum* could result in greater induction following infection leading to greater resistance.

In this work, a novel VIGS-vector with ToMoV was developed, which did not produce severe viral symptoms and was able to silence genes in *S. peruvianum*. With this vector, it was possible to determine that SCEI is important in the defense of *S. peruvianum* against *Cmm*, possibly because SCEI impacted ETI through the effects of sumoylation on transcription factors, like WRKYs, and/or the production of the defense hormones, like SA. This study only examined silencing of *SCE*I in *S. peruvianum* in its relationship to innate immunity to *Cmm*, but *SCE*I may also play roles in resistance to other diseases or pests. For example, *S. peruvianum* has resistance against root-knot nematode (Yaghoobi et al., 2005) and tomato leaf curl virus (Anbinder et al., 2009). Therefore, altering expression of *SCE*I may be a strategy to increase resistance not only against *Cmm* but also against several other diseases. Based on our findings, one biotechnological approach to improve the *Cmm* resistance on commercial tomato cultivars is the overexpression of *SCE*I gene either using constitutive promoters or the promoter region of the *SCE*I gene from *S. peruvianum* which may activate the SA signaling pathway following infection of *Cmm*, resulting in an enhanced resistance to this bacterial disease.

## Author contributions

ME, BB, GA, JL, and AA design the study. ME, BB, and MI collected the data. ME, BB, GA, AA, and BB analyzed the data. ME, BB, GA, AA, PG, and AR interpreted the data. ME wrote the first draft and all co-authors contributed substantially to revisions.

### Conflict of interest statement

The authors declare that the research was conducted in the absence of any commercial or financial relationships that could be construed as a potential conflict of interest.
