# Supplementary material for: Evaluation of a SUMO E2 Conjugating Enzyme Involved in Resistance to Clavibacter michiganensis Subsp. michiganensis in Solanum peruvianum, Through a Tomato Mottle Virus VIGS Assay
Source: Front Plant Sci. 2015 Dec 17;6:1019. doi: 10.3389/fpls.2015.01019 (PMC4681775; doi:10.3389/fpls.2015.01019)

**Evaluation of a SUMO E2 conjugating enzyme involved in resistance to *Clavibacter michiganensis* subsp*. michiganensis* in *Solanum peruvianum,* through a tomato mottle virus VIGS assay.**

Mayra Janeth Esparza-Araiza^1^, Bernardo Bañuelos-Hernández1, Gerardo Rafael Argüello-Astorga^1^, José Pablo Lara-Ávila^2^, Paul H. Goodwin^3^, María Isabel Isordia-Jasso^1^, Rosalba Castillo-Collazo^1^, Alejandra Rougon-Cardoso^4^, Ángel Gabriel Alpuche-Solís^*^

^1^ IPICYT, División Biología Molecular, Instituto Potosino de Investigación Científica y Tecnológica A.C., Camino a la Presa San José 2055, Lomas 4ta. Sección, 78216, San Luis Potosí, S.L.P. México.

^2^ Facultad de Agronomía y Veterinaria, Universidad Autónoma de San Luis, Km 14.5 Carretera San Luis Potosí, Matehuala, Ejido Palma de La Cruz, Soledad de Graciano Sánchez 78321, San Luis Potosí. S.L.P. México

^3^ School of Environmental Sciences, University of Guelph, 50 Stone Road East, Ontario N1G 2W1, Canada.

^4^ Laboratory of Agrogenomic Sciences, Universidad Nacional Autónoma de México (UNAM), ENES-León, 37684, Guanajuato, México.

^*^Corresponding author:

Á. G. Alpuche-Solís, IPICYT, División Biología Molecular, Instituto Potosino de Investigación Científica y Tecnológica A.C., Camino a la Presa San José 2055, Lomas 4ta. Sección, 78216, San Luis Potosí, S.L.P. México. e-mail: alpuche@ipicyt.edu.mx

**Figure 1S. Alignment of *SCE*I proteins of *Solanum* sp.**

Fragment_*SCE*I, corresponds to fragment isolated by Lara-Ávila et al. (2012), cloned in ToMoVΔCP and silenced. Solyc_ch02_g093110, Solyc_ch03_g044260, Solyc_ch04_g078620, Solyc_ch12_g088680 correspond to SUMO conjugating enzyme (SCE) paralogs in *S. lycopersicum.* * (asterisk) indicates positions which have a single, fully conserved residue. : (colon) indicates conservation between groups of strongly similar properties - scoring > 0.5 in the Gonnet PAM 250 matrix. . (period) indicates conservation between groups of weakly similar properties - scoring = < 0.5 in the Gonnet PAM 250 matrix. The conserved catalytic Cys is indicated by the arrowhead, and the ubiquitin-conjugating enzyme signature is underlined.


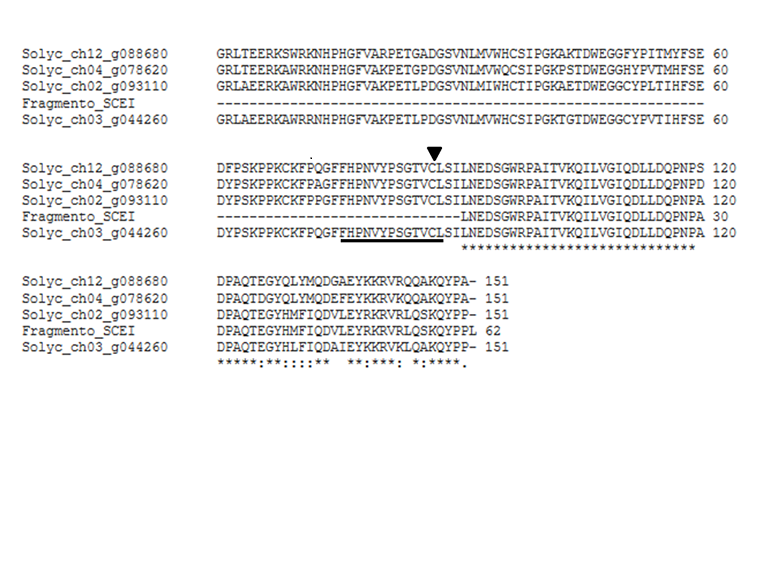

Supplement: Supplementary file 1 [file DataSheet1.doc]
